# Research Progress on Common Sports Injuries Among Youth Ice Hockey Players and Prevention Strategies: A Narrative Review

**DOI:** 10.3390/sports13120449

**Published:** 2025-12-11

**Authors:** Yalin Zheng, Yawen Liu, Yimei Chen, Jie Cao, Enyuan Chen, Hongjing Pan, Peng Huang

**Affiliations:** 1 School of Sports Medicine and Rehabilitation, Beijing Sport University, No. 48 Xinxi Road, Haidian District, Beijing 100084, China; zhengyl@bsu.edu.cn (Y.Z.); 2023210395@bsu.edu.cn (Y.L.); 2020012093@bsu.edu.cn (Y.C.); 2025210383@bsu.edu.cn (J.C.); cey2021011232@bsu.edu.cn (E.C.); 2 Department of Physical Education and Research, Beijing Foreign Studies University, No. 2 Xisanhuan North Road, Haidian District, Beijing 100084, China

**Keywords:** ice hockey, common sports injuries, prevention strategies, youth players

## Abstract

Background: Ice hockey is a high-intensity collision sport with one of the highest injury rates among youth team sports. Despite advanced protective equipment, youth athletes remain particularly vulnerable due to their unique physiological and psychological characteristics. Objective: This narrative review aims to synthesise the current evidence on the epidemiology, risk factors, and prevention strategies for common sports injuries in youth ice hockey players. Methods: We conducted a comprehensive literature search across PubMed, Web of Science, Scopus, and the Cochrane Library for publications between August 2015 and August 2025 using an iterative process and manual reference screening to identify relevant studies. Result: The evidence indicates that injury rates are notably high, ranging from 11.7 to 34.4 per 1000 athlete-hours. Concussions and upper/lower limb injuries are most prevalent. Body checking is the most significant modifiable risk factor associated with a threefold increase in concussion incidence. Policy interventions prohibiting body checking have demonstrated substantial benefits, leading to a 50–70% reduction in injury rates and a 57–60% reduction in concussions. Furthermore, the use of full-face protection was associated with a fourfold reduction in the risk of facial and dental injuries. Specialised preparatory activities and neuromuscular training, as well as comprehensive safety and rules training for players and coaches, can reduce the risk of injury in youth hockey players. Conclusion: This review underscores that effective injury prevention in youth ice hockey requires multi-faceted strategies focused on policy changes and proper equipment. Future work should focus on developing personalised prevention models, establishing youth-specific equipment standards, and enhancing safety awareness.

## 1. Introduction

Ice hockey is a competitive team sport played on ice using skates and sticks. Modern ice hockey originated in Canada, where participants wearing skates and wielding sticks pursued and struck a circular puck across frozen lakes, aiming for two upright wooden posts. With no limit on the number of players, this game is regarded as the prototype of modern ice hockey [1]. Subsequently, the rules evolved and spread globally. Ice hockey combines high-intensity intermittent skating and rapid acceleration and deceleration with frequent physical contact. Players rely on high-speed skating while possessing the ability to accelerate sharply, brake abruptly, change direction, and skate backwards. They must also control the puck and execute tactical plays to deliver precise passes and powerful shots [2]. The game features rapidly shifting attack and defence, intense physical confrontation, and possesses tremendous visual impact. Statistics indicate that global participation in ice hockey has risen steadily over the past decade. Annually, over 1.6 million male athletes from 79 countries and regions engage in the sport [3]. Furthermore, numerous nations are introducing ice hockey through school programmes, youth-specific leagues, and introductory winter sports activities, enabling increasing numbers of young people to discover and develop a passion for the game.

The rapid advancement of technology has seen athletes equipped with specialised protective gear; nevertheless, research indicates that ice hockey remains one of the team sports with the highest injury rates. The particularity of youth athletes lies in their significant physiological and psychological differences from adult athletes, which are associated with an increased risk/are considered risk factors for sports-related injuries [4]. Physiologically, youth athletes are in a unique stage of growth and development, encompassing body composition, metabolic and hormonal fluctuations, organ system maturation, and nutritional aspects [5]. Notably, the epiphyseal growth plates in youth remain open. Prolonged training involving excessive external impacts, shear forces, avulsion forces, or compressive forces can readily cause growth plate injuries, potentially leading to chondrocyte damage, premature closure of the growth plate, and skeletal growth disorders [6]. Furthermore, imbalances in muscle and tendon development are more pronounced in youth. A growing body of research indicates that muscle and tendon adaptations during training do not necessarily occur in a uniform manner, particularly among youth athletes. Growth and development exert additional stimulatory effects on the maturation of the muscle-tendon unit, potentially exacerbating disparities between muscle strength and tendon stiffness [7]. Moreover, youth athletes are in a critical developmental phase for coordination and balance. Their limited body control when executing complex, high-difficulty technical movements makes them prone to postural deviations and inefficient force generation, leading to the formation of incorrect movement patterns that may further elevate injury risk [8].

Moreover, research indicates that the incidence of mental disorders among youth is rising rapidly, with approximately half of all mental health conditions emerging before the age of 18, and adolescence representing the peak onset period [9]. Although physical activity is often recognised as beneficial for mental well-being, entry into professional training systems subjects young athletes to high-intensity, high-frequency training loads and an emphasis on competitive outcomes. This places them within a highly competitive environment, facing multifaceted pressures from parents, peers, coaches, competitions, and society [10]. Excessive psychological stress is linked to impaired decision-making abilities and athletic performance and is associated with anxiety, depression, and an elevated risk of injury.

In summary, we should place greater emphasis on the unique requirements of young athletes. By addressing their specific needs and accounting for their physical development characteristics, we can devise safer and more effective training programmes to foster their healthy growth and all-around development. This will empower them to realise their athletic potential and elevate their competitive performance. Although previous reviews have provided a foundation for understanding injuries in youth ice hockey, the rapid evolution of the sport, including major rule changes, the current state of epidemiology, and advances in protective equipment and neuromuscular training strategies, urgently require further, more comprehensive and integrated analyses. In addition, existing reviews suffer from outdated content and lack a multidimensional critical analysis of epidemiology, modifiable risk factors, and evidence-based preventive measures. Based on this, this narrative review comprehensively categorises common injury types among youth ice hockey players, conducts an in-depth analysis of injury mechanisms and risk factors, and summarises existing injury prevention strategies for the sport. This aims to provide novel approaches for injury risk assessment and sports injury prevention, thereby promoting the sustainable development of ice hockey and offering theoretical foundations and practical guidance for fields including sports injury prevention and youth sports health.

## 2. Methods

This narrative review synthesises the current understanding of Common Sports Injuries Among Youth Ice Hockey Players and Prevention Strategies. The literature search was conducted using PubMed, Web of Science, Scopus databases, and Cochrane Library, covering publications from August 2015 to August 2025. Keywords such as “Ice hockey”, “Sports Injuries”, “Prevention Strategies and rehabilitation”, and “Youth Players” were used. The selection of included articles was iterative, aiming to provide a critical overview of the field. Manual searching of reference lists was also employed to ensure the inclusion of seminal and influential studies.

## 3. Analysis of Common Sports Injuries Among Youth Ice Hockey Players

### 3.1. Incidence of Injury

Due to high-speed skating and rules permitting body checks, injury rates among youth ice hockey players remain persistently high [11]. Over half of ice hockey players attending US emergency departments in the 2001–2002 period were adolescents, with those aged 12–17 accounting for 47% [12]. In recent years, the number of 9–14-year-old youth ice hockey players treated for injuries in US emergency departments has also been steadily increasing [13,14].

A systematic review indicated that the overall injury rate among youth ice hockey players ranged from 11.7 to 34.4 per 1000 player-hours [14]. Much of this wide variation stems from the methodological heterogeneity of the included studies, such as differences in injury definitions, monitoring methods, and levels of participation. In contrast, in highly standardised settings, such as the 2009–2016 World Junior Ice Hockey Championships, injury rates were as high as 39.8 injuries per 1000 game hours [15], which underscores the high-intensity nature of competition at the elite level. In conclusion, injury rates vary by age group and playing rules, and it is important to consider the context and methodology when interpreting these data. Injury rates among youth ice hockey players vary across age groups and competition rules. Research indicates that Midget (16/17 years) players face higher injury risks than Pee Wee (11/12 years) and Bantam (13/14 years) players [16]. Following Canada’s 2013 policy change prohibiting bodychecking in Pee Wee competitions, the overall injury incidence in this age group decreased from 4.37 per 1000 player-game hours to 2.16 per 1000 player-game hours—a 50% reduction [17]. Although such before-and-after observational studies cannot completely rule out other confounding factors, the large decrease in injury rates constitutes strong evidence in favour of the policy. This finding is consistent with other studies that have concluded that leagues that prohibit body punching have lower injury rates than leagues that allow it [18,19]. Together, this evidence suggests that rule changes are one of the most effective interventions for reducing youth hockey injuries. In addition, injuries occur more frequently during matches than in training sessions [20]. The injury rate during matches is over eight times higher than during training sessions, and can be as much as 25 times higher [15]. Most studies found male adolescent ice hockey players had higher injury rates than female players, though some studies reported no gender difference in injury rates [13,16,21,22].

As the previous data indicate, the incidence of injury in youth ice hockey is difficult to quantify precisely because a variety of factors influence it. The main limitations of the available evidence are: retrospective and prospective reports, the selection of ice hockey player samples, the sources of injury data collection, potential bias in athlete self-reporting or physician diagnosis, varying methods for measuring athlete participation and calculating injuries, and, most critically, there is a lack of uniformity in the definition of impairment. These methodological inconsistencies have a direct impact on the accuracy and comparability of the data, and therefore, extra caution is needed when comparing different studies [23]. Furthermore, ongoing modifications to ice hockey rules and policies present significant challenges in establishing universally applicable injury probability conclusions. Future research may necessitate the development of more authoritative and standardised databases. These should incorporate rigorous professional assessment of athlete injuries, enabling comprehensive and accurate data collection to facilitate subsequent analysis of injury probabilities and mechanisms.

### 3.2. Injury Location

The head and face are the most commonly injured areas among youth ice hockey players, followed by the upper or lower limbs, though this remains a point of contention across different studies. The least frequently affected regions are the spine and trunk [20,24]. Subtle variations in injury locations may also exist across different age cohorts of youth. Injury data from the 2006–2015 World Junior Ice Hockey Championships for the WJ U20 and WJ U18 categories revealed that facial injuries were most prevalent in the WJ U20 tournament, whereas shoulder injuries were most common in the WJ U18 competition [15]. One study indicated that head and spinal injuries were prevalent among players under 14 years old, whereas facial injuries were more common among athletes aged 15 and above [25]. Differences in injury locations may also relate to match or training contexts. Injury surveillance of American high school boys’ ice hockey from the 2008–2009 period to the 2013–2014 period found that the head and face were most frequently injured during matches, the shoulders during matches, and thighs/hips and knees during training sessions [20].

#### 3.2.1. Head and Face

The head and face are the most common sites of injury among youth ice hockey players, including concussions, facial lacerations, and dental injuries. A concussion is the most common injury following a head impact, leading to fatigue, headaches, distractibility, and other symptoms. The aggregate incidence of concussion among youth ice hockey players can reach 9–18 concussions per 100 players, or 1.3–1.6 per 1000 player-hours, with a minority of athletes experiencing recurrent concussions [19,26,27]. Before Canada’s policy change on bodychecking, studies indicated Pee Wee concussion rates could reach 2.31 per 1000 player-hours. Growing evidence indicates that prohibiting bodychecking in youth competitions correlates with reduced injuries [28]. Illegal bodychecking manoeuvres cause severe injuries, and no-bodychecking policies reduced concussion rates by 50–60% in 11–12-year-old Pee Wee teams [17,18]. Among youth athletes of different age groups, younger players exhibit higher concussion rates than their older counterparts [26,29].

In the 1960s, following the mandatory introduction of helmets with face masks for youth athletes in the United States, the incidence of facial injuries gradually declined. Athletes wearing half-face masks were over four times more likely to sustain facial lacerations and dental injuries than those wearing full-face masks [30,31]. The majority of facial injuries were lacerations, with studies indicating that athletes under 18 sustained significantly more lacerations than those aged 18–24 [15]. Facial injuries during matches were markedly higher than during training sessions, with the risk of facial injury in matches reaching up to 60 times that during training [20]. Facial lacerations are frequently associated with ice hockey sticks and may also relate to the facial protective equipment worn. Wearing only goggles increases the likelihood of facial lacerations, whereas full-face protective gear reduces the incidence of head and neck lacerations in adult ice hockey players [32].

#### 3.2.2. Upper Limb

Compared to adult players, youth ice hockey athletes experience a higher proportion of upper limb injuries [12]. The majority of upper limb injuries occur in the shoulder region, followed by the wrist and elbow [4]. A study analysing 760 Finnish ice hockey players found that body checks caused 76% of shoulder injuries, 55% of elbow injuries, and 45% of distal extremity injuries, with 38% of these being severe. Upper limb injuries may result from multiple injury mechanisms, with both body checks and stick strikes contributing to upper limb trauma. Studies have observed that the incidence of upper limb injuries increases with age within the 12–19 age range [33,34].

Body checks and stick strikes account for a significant proportion of shoulder injuries [35]. Acromioclavicular joint injuries represent the most common shoulder injury, typically resulting from forceful impact with an opposing player or the ice surface. This may involve tearing of intra-articular ligaments, leading to joint pain, swelling, and restricted range of motion. Youths possess weaker shoulder musculature compared to adults, rendering them more susceptible to injury during collisions [15,36]. Concurrently, the clavicle faces a high fracture risk during severe shoulder impacts, potentially linked to skeletal immaturity [37]. Studies observed that among American high school athletes from 2008 to 2017, male ice hockey players had the highest incidence of clavicle fractures, followed by field hockey, football, and wrestling [38]. The time loss due to a clavicle fracture may extend to 8–10 weeks, with the precise recovery period potentially influenced by treatment methods and other factors, rendering post-injury rehabilitation a complex undertaking [39].

Elbow injuries in ice hockey players may stem from falls, collisions, or striking motions. Elbow contact with the ice or forceful impacts can cause fractures at the distal humerus, olecranon, or radial head [40]. Direct blows may result in contusions or lacerations, with traumatic olecranon bursitis being a typical elbow contusion [41]. Repetitive striking motions in athletes may cause ulnar collateral ligament injuries. The sustained stress from repeated impacts induces microtears in the ligament, potentially progressing to complete rupture [42].

Injuries to the hand and wrist are relatively common, encompassing ligament damage, contusions, and fractures [33]. ‘Gamekeeper’s thumb’ denotes ulnar collateral ligament injury of the thumb [43], a sport-specific injury in ice hockey where excessive thumb abduction during falls while holding the stick causes ligament damage. Contusions of the forearm, wrist, and hand are also frequent, typically occurring during impacts [34]. During body checks and stick strikes, injuries such as distal radius fractures, carpal fractures, and metacarpal fractures may occur. The tip of the hockey stick contacts the ulnar carpal bones and repeatedly strikes the hamate bone during play, posing a significant risk of hamate fractures. Repetitive stress on the ulnar side may also cause triangular fibrocartilage complex (TFCC) injuries and ulnar collateral ligament (UCL) damage, though statistical data on these injuries is limited [44,45].

#### 3.2.3. Lower Limb

Lower limb injuries in youth ice hockey primarily encompass hip/buttock/groin pain, knee ligament injuries, and ankle sprains. Hip and groin injuries are predominantly caused by overuse, whilst knee and ankle injuries are mostly acute sprains [24].

Research indicates that approximately 9–11% of youth ice hockey players experience hip/buttock/groin pain [16]. One study comparing hip injury rates among athletes aged 11–12 to those over 20 found that 75.5% of hip injuries occurred in youth athletes aged 15–20, with the majority being non-contact injuries. Contact-related injuries, involving collisions with opponents or the ice surface, most commonly manifest as strains and contusions. The hip joint is particularly vulnerable to injury during flexion and rotational movements [23].

Groin pain is a highly prevalent symptom among youth ice hockey players, with the most common mechanisms being non-contact injury and overuse [46]. Youth ice hockey players frequently experience adductor strain, with all six muscles within the adductor group potentially affected. This relates to the specific movements of ice hockey. To maintain increased stride length during high-speed skating, the hip joint requires greater abduction. The repetitive eccentric contraction of the adductors during this abduction may lead to strain, subsequently causing restricted hip abduction and pain [47]. Research has found that athletes who specialise in ice hockey before secondary school experience more severe hip and groin dysfunction, which can even impact their quality of life and activities of daily living [48]. Another potential cause of groin pain is femoroacetabular impingement. Research has found that youth ice hockey players exhibit a greater hip alpha angle associated with femoral acetabular impingement [49,50]. Additionally, the goalkeeper’s distinctive butterfly kneeling position requires internal rotation of the femur, further increasing impingement risk. The cam-type femoroacetabular impingement is more prevalent among ice hockey players [51]. Although current research has paid relatively little attention to sports injuries among youth ice hockey goalkeepers, the specific movements involved and the heightened risk of injury suggest that greater emphasis and observation should be directed towards players in this position. Analysing the mechanisms of injury could help reduce their incidence, thereby extending players’ career longevity.

The medial collateral ligament is one of the most common sites of injury in the knee joint among youth ice hockey players, and also one of the ligaments most frequently injured in the knee joint [52]. Body checks or changing direction upon landing cause an outward rotational force on the knee joint, leading to medial collateral ligament injury, which may present with symptoms such as knee swelling [53]. Injuries to the anterior cruciate ligament and lateral collateral ligament are relatively uncommon in ice hockey [39], owing to the sport’s specific injury mechanisms and biomechanics, with most injuries occurring during valgus stress rather than varus stress.

Among youth ice hockey players, high ankle sprains are as prevalent as low ankle injuries. Injuries to the distal joint of the tibia and fibula are termed high ankle sprains, also known as syndesmosis injuries [51]. The occurrence of high ankle sprains correlates with the characteristics of ice hockey. Players must wear rigid ice skates, which restrict ankle mobility. Forces generated by twisting motions during high-speed skating can cause ligament damage or even separation of the distal ends of the tibia and fibula, resulting in high ankle sprains [54]. Additionally, when athletes are adapting to new ice skates, they may experience skate bite in the ankle region. An excessively rigid skate tongue exerts pressure on the anterior aspect of the ankle. Repeated dorsiflexion to counteract this pressure may cause inflammation of the tibialis anterior muscle [55].

#### 3.2.4. Spine

Research indicates that between 1943 and 1999, Canadian ice hockey players reported a total of 271 spinal injuries, with players aged 16 to 20 accounting for 49% of cases. The injury rate has shown a gradual decline over the years [56]. Between 2006 and 2011, Canadian ice hockey players sustained 44 spinal injuries, four of which were severe. Male athletes aged 11–20 accounted for the highest number of injuries. The majority of injuries occurred in the cervical region, with approximately three-quarters resulting in neurological deficits. Furthermore, about one-quarter of injuries led to the complete loss of all motor function below the level of injury [57]. However, the incidence of spinal injuries has continued to decline in recent years, potentially due to the introduction of rules prohibiting body checks from behind [58]. Whether the introduction of head and face protection has caused greater harm to the spine remains controversial. Some studies suggest that protective gear may give athletes a false sense of security, leading to more aggressive play and increasing the likelihood of neck injuries. Conversely, other research indicates that head protection has no impact on neck injuries [32,59].

## 4. Analysis of Risk Factors for Sports Injuries Among Youth Ice Hockey Players

Ice hockey is a highly competitive sport that demands speed, technical skill, balance, and physical fitness. Due to its high-speed skating and intense physical contact, coupled with the unique physical and psychological development characteristics of youth, young ice hockey players face a heightened risk of sports injuries during training and competition. Understanding the risk factors for sports injuries in youth ice hockey players assists clinicians in both treatment and injury prevention. Common risk factors primarily include physical contact, competition format, competition level, age, gender, body weight, player position, and protective equipment.

### 4.1. Performance Characteristics

#### 4.1.1. Body Checking

Due to the nature of the game’s rules, body checking represents one of the primary risk factors for injury in youth ice hockey players. Compared to leagues prohibiting physical contact, athletes competing in leagues permitting body contact experience have a significantly higher overall injury risk, and a threefold higher rate of concussion [19]. Research indicates that concussion rates decrease by 58% under no-contact regulations, robustly validating the efficacy of policies prohibiting physical contact for children and youth ice hockey players [11,18,60]. A systematic review demonstrated that participation in contact-permitted leagues within the under-18 age group resulted in a 2.45-fold increase in match injuries and a 1.71-fold increase in concussion rates. Among all potential risk factors, physical contact policies have been identified as the single most consistent factor for injuries and concussions [14]. Another landmark study demonstrated that such policies in under-13 age groups led to a 3.26-fold rise in match injuries and a 3.88-fold increase in concussions [19]. These findings collectively indicate that physical contact rules constitute the most critical risk factor for injuries, particularly concussions, among youth ice hockey players, with younger age groups facing heightened injury risks. This underscores the paramount importance of strictly enforcing non-contact rules within these age cohorts.

#### 4.1.2. Competition Level

Within the realm of youth ice hockey, a significant positive correlation exists between competition level and the risk of injury among young athletes. As the level of competition increases, so too does the intensity and physicality of play, a phenomenon termed competitive stress [61]. High levels of competitive stress are associated not only with an increase in the frequency of physical contact on the ice but also with more intense physical confrontations, which in turn are linked to a higher likelihood of injury [62].

### 4.2. Individual Athlete Factors

#### 4.2.1. Age and Relative Age

Youth ice hockey categories are primarily divided by age group into Under-18 (15–17 years; formerly known as Midget), Under-15 (13–14 years; formerly known as Bantam), Under-13 (11–12 years; formerly known as Pee Wee), Under-11 (10–11 years; formerly known as Atom), and Under-9 (8–9 years; formerly known as Novice). Age serves as a potential risk factor for injuries among youth ice hockey players, with injury rates generally increasing with age [16]. Whilst these studies provide compelling evidence, some research suggests no significant increase in injury risk for older youth groups, leaving the matter contentious. Roberts et al. [63] reported a relative risk of injury of 0.71 for Bantam compared to Pee Wee. Similarly, Williamson et al. [64] found older players to be at lower risk than younger ones. However, Wattie et al. [65] demonstrated no significant difference in injury risk across age groups. These factors may substantially obscure or modify the true relationship between age and injury risk [66]. Consequently, future research employing more refined study designs is essential to elucidate this relationship further. This is crucial for developing age-specific risk management and injury prevention strategies aligned with athletes’ developmental stages.

#### 4.2.2. Gender

For female athletes, menstrual history represents a potential risk factor. At the PeeWee level, players who commence menstruation at the start of the season face a fourfold increase in injury risk compared to those who have not yet begun menstruating [22]. Men are more prone to shoulder dislocations and overall shoulder injuries, whereas women are more susceptible to wrist injuries and wrist strains or sprains [67]. Wilkie et al. emphasised the impact of gender-specific biomechanical differences on ice hockey stick-swing mechanics, with males exhibiting greater trunk flexion and lead shoulder flexion, while females demonstrated increased anterior shoulder abduction, elbow flexion, and trail wrist extension during the backswing and downswing phases [68]. It is therefore essential to incorporate gender differences into the analysis of risk factors for sports injuries, as this provides a foundation for developing targeted injury prevention strategies and enhancing the safety of ice hockey players.

#### 4.2.3. Player Position and Skill Level

Player position has been identified as a risk factor for injury among youth ice hockey players in three relevant studies. Roberts et al. [56] found forwards to be at higher risk of injury than defencemen and goalkeepers, and Stuart et al. [69] reported that the RR for injury among defencemen was 2.18 times that of forwards. However, multiple studies indicate that goalkeepers have the lowest injury rates compared to other player positions.

Emery and Meeuwisse et al. [16] observed that the highest-skilled players in the Pee Wee age group bore the greatest injury risk, though no significant increase in risk was associated with skill level in other age groups. Conversely, the remaining two studies found that risk increased with rising skill levels within age groups [65,70]. Existing research on player position and technical level as injury risk factors presents a complex and inconsistent picture. Regarding player position, the conclusion that goalkeepers face the lowest risk is relatively consistent. Future studies should undertake prospective cohort research, further integrating performance analysis—such as through video analysis or wearable devices—to precisely quantify activity loads and contact events for players in different positions, thereby revealing the specific mechanisms underlying injury occurrence.

#### 4.2.4. Injury History

Players with a prior history of injury or concussion are typically identified as being at heightened risk of injury [71]. This association may be explained by multiple mechanisms: firstly, the initial injury may cause persistent deficits in physical function (such as neuromuscular control, proprioception, and balance), leaving the athlete in a biomechanically vulnerable state upon return to sport [72,73]; secondly, premature return to competition before full recovery substantially increases the risk of re-injury to the original site or compensatory injuries to other areas [73,74]; thirdly, psychological factors cannot be overlooked, as fear of re-injury may alter athletes’ decision-making and movement patterns, thereby increasing risk [75]. However, further research is needed to better understand the complex relationships and internal connections within youth ice hockey and other sports and to investigate whether the relationship between prior injury history and re-injury risk follows the same pattern in other youth contact sports, such as football and basketball.

### 4.3. Equipment and Protection Factors

#### Personal Protective Equipment

Observational and case–control studies have generally shown that properly worn helmets reduce the severity of concussions and the duration of symptoms [76]. Inadequate helmet fit may influence the likelihood of youth ice hockey players sustaining concussions [27]. Post et al. [77] developed an adult ice hockey helmet assessment methodology, demonstrating that rotational velocity and rotational acceleration can be identified as useful performance metrics representing concussion risk levels. However, an important limitation in this field is that many of the biomechanical testing and evaluation standards for helmet protection have been developed based on adult data. Research and standards for helmets that specifically address the anatomical structure of the youth head and the biomechanical properties of impact are still missing, and this is a critical gap that needs to be filled in the future.

The benefits of wearing a face mask in ice hockey are to prevent dental, facial, and ocular injuries. Substantial evidence indicates that full-face protection (FFP) reduces both the number and risk of head and facial injuries in ice hockey compared to partial-face protection (PFP) without facial protection. Whilst PFP offers less protection than FFP, it appears to reduce risk more than no protection at all [78]. Evidence suggests that FFP in ice hockey mitigates concussion severity, with players wearing PFP requiring longer recovery times than those wearing full-face masks when concussions occur [79]. Injuries to the upper face are observed to be less common among masked players. Following the National Hockey League’s(NHL) mandatory mask requirement, the average number of craniomaxillofacial and upper-face injuries per season decreased significantly [80].

A nested case–control study involving 315 participants demonstrated that the use of mouthguards in ice hockey games provides significant protective effects [81], associated with lower rates and odds of concussion occurrence, with particularly pronounced protective effects in the youth cohort [82]. However, some studies indicate that wearing a mouthguard during injury is not associated with reduced acute or subacute symptoms following sports-related concussion, compared to children and youth who did not wear one. Nevertheless, we continue to encourage athletes to wear mouthguards during sports, as the overwhelming evidence supports their effectiveness in preventing dental injuries [83].

In summary, existing evidence consistently indicates that correctly worn protective equipment (helmets, full-face masks, and mouthguards) plays a crucial role in mitigating concussion severity, reducing symptom duration, and preventing maxillofacial injuries. However, significant limitations remain in this field of research. Future studies should focus on establishing standards for the personalised selection, fitting and efficacy evaluation of protective equipment for youth ice hockey, thereby providing a theoretical basis for developing more targeted protective strategies.

## 5. Prevention Strategies for Common Sports Injuries Among Youth Ice Hockey Players

### 5.1. Fair Play and Rule Amendments

Fair Play Points (FPP) establish a clear system of rewards and penalties, recognising conduct demonstrating sporting spirit while penalising breaches of ethical standards. When a team exceeds the predetermined Penalty Minutes per Match (PIMS) threshold in any fixture, its FPP are forfeited. When a team earns FPP for sporting conduct, these points are added to the two points awarded for a win or the single point for a draw. In practice, this has demonstrated a significant injury prevention effect [84] (Table 1). Research findings indicate that implementing FPP effectively reduces penalty minutes, the incidence of serious fouls, and injury rates during matches. In Minnesota’s youth ice hockey leagues, combining FPP with a ban on body-checking rules led to a significant decrease in the frequency of check-from-behind incidents and head-to-head collisions [85,86].

Optimising the rule system and standardising officiating criteria are crucial aspects of preventing sports injuries. Epidemiological studies reveal marked gender differences: male athletes predominantly sustain injuries to the head, neck, and upper limbs, with shoulder injuries frequently causing permanent damage—a direct consequence of permitted physical collisions. Female athletes, conversely, face greater risks of knee injuries and concussions, reflecting how differing playing styles and rule applications profoundly influence injury patterns [87]. Consequently, major ice hockey associations have actively implemented stricter rule revisions and officiating standards, particularly adopting a ‘zero-tolerance’ policy towards head-on collisions and charging from behind [88]. Once confirmed, such dangerous actions will result in penalties for players, including removal from the game and suspension. Physical contact is entirely prohibited for players aged 12 and under; older players are permitted limited physical contact when contesting possession; and all youth players are explicitly barred from dangerous actions such as charging an unguarded opponent from behind or engaging in intimidating behaviour. These measures have effectively curbed the occurrence of deliberate foul play. In a cohort study involving 688 NHL players with concussions, a reduction in concussion incidence following direct lateral head impacts was observed after the implementation of relevant regulations. The rate of concussions resulting from lateral head impacts decreased by 0.6 per 100 games, representing an 18.8 percentage point reduction [58]. Following the implementation of rules prohibiting body checks in non-elite 15–17 age group competitions, a 70% reduction in injury rates and a 57% reduction in concussion rates were observed [11].

In summary, fair play initiatives and rule revisions have proven effective strategies for reducing injury rates in youth ice hockey. However, there remains a lack of in-depth analysis regarding how implementation details—such as the rigour of rule enforcement and consistency in officiating decisions—specifically influence prevention outcomes. Future research should focus on further exploring optimal injury prevention regulations.

### 5.2. Research and Development of Protective Equipment

Ice hockey players must wear standard-compliant protective gear (including helmets, shoulder pads, elbow pads, knee pads, etc.) and regularly inspect the integrity of their equipment (Table 1). Scientifically appropriate gear configurations are crucial safeguards for player safety, effectively cushioning impacts while significantly reducing both the likelihood and severity of injuries. With technological advancements, protective equipment in ice hockey is continually evolving through material innovation, intelligent monitoring systems, and personalised designs to enhance player safety.

Boorady et al. [89] claimed that poor fitting not only diminishes the protective function of the equipment but also exacerbates the problem of the pad shifting. However, to date, most tibial and elbow protectors have employed elastic bands and Velcro fastenings to secure them from the rear. As protective equipment may become displaced, careful consideration must be given to potential solutions concerning their technical design. Moreover, the shape and fit of helmets, along with the proportions of shin guards, are also crucial in injury prevention [27]. Consequently, developing protective equipment tailored to Asian body measurements is essential for injury prevention.

In terms of material innovation, D3O, an impact-resistant material, is hailed as one of the top ten future materials. In its normal state, D3O remains soft and flexible, allowing sufficient joint mobility. However, upon impact, it locks itself to dissipate energy [90]. Research indicates it can reduce peak collision forces by over 30%. Moreover, the intelligent protection system employs embedded sensors to monitor acceleration, angular velocity, and other parameters in real time, issuing alerts for abnormal collisions and excessive fatigue among athletes. The smart helmet precisely records head collision parameters, providing a basis for early concussion detection and further clarifying the characteristics of head impact exposure [59]. Future research should employ biomechanical modelling to further evaluate and compare the protective efficacy of novel protective equipment materials. Furthermore, specific testing standards for youth protective gear should be developed, taking into account the unique characteristics of sports injuries in young athletes. This aims to enhance athlete protection and prolong their sporting careers.

### 5.3. Specialised Preparatory Activities and Neuromuscular Training

Strategies for preventing sports injuries in adolescent ice hockey players have gradually evolved into a multidimensional integrated system centred on neuromuscular training (Table 1). Training should focus on enhancing muscle strength and flexibility in injury-prone areas. This involves improving hip joint mobility, strengthening adductor muscles and soft tissue flexibility, combined with activation of abductor/external rotator muscles and core stability training. These measures reduce muscular load during activity, thereby minimising injury risk. Concurrently, pre-season functional assessments and regular in-season muscle strength monitoring help prevent injuries such as medial collateral ligament strains in the knee [91]. Furthermore, training necessitates thorough warm-ups, scientifically combining aerobic and anaerobic exercises, with careful load management and progressive development to avoid overtraining, thereby comprehensively enhancing physical conditioning.

Neuromuscular training has emerged as a key approach in injury prevention. The training programme, developed to address non-contact injuries common during lateral movement, landing/stopping, and acceleration/deceleration phases, is grounded in the RAMP principle (Raise, Activate and Mobilise, Potentiate). This scientific framework progressively elevates body temperature, activates key muscle groups, mobilises major joints, and simulates match intensity. It has been demonstrated to effectively reduce injury risks stemming from inadequate neuromuscular control, optimise biomechanics, diminish landing impact forces, and enhance athletic performance [92,93]. In the realm of concussion prevention, the neuromuscular training programme innovatively incorporates a ‘neck control and endurance’ component [94]. Enhancing neck muscle strength and endurance improves head stability and reduces the risk of concussion from collisions during athletic activity [95]. This programme integrates neuromuscular training with ice hockey-specific techniques, employing a combined off-ice and on-ice approach. It incorporates hockey-specific movements for dynamic mobility and stationary stability training. Initial practice findings indicate favourable applicability and acceptance.

### 5.4. Education and Management

Both athletes and coaches require comprehensive safety and rule training (Table 1). For youth ice hockey players, emphasis should be placed on enhancing their self-protection awareness, ensuring they fully comprehend the specific characteristics of this age group and the importance of wearing appropriate protective gear. Adequate warm-ups before training and matches must be ensured to reduce injury rates. Concurrently, they must comprehend the dangers of head collisions and checks from behind, learning to recognise and appropriately manage related injuries. Whilst secondary concussion prevention education programmes may enhance their cognitive understanding and safety awareness to some extent, most research indicates the current evidence base remains insufficient. Athletes often struggle to translate acquired knowledge into practical action [96]. Therefore, alongside advancing educational initiatives, further research is required to develop effective interventions that enhance athletes’ implementation of learned behaviours.

Research indicates that mental health remains a sensitive issue for ice hockey players, as barriers to seeking support include feelings of shame, a deeply ingrained hockey culture, a lack of mental health literacy, and negative past experiences of seeking help [97]. Coaches continue to underemphasise this aspect. Moreover, the influence of coaches and the atmosphere they create within athletes’ lives is also significant. In a recent study [98], young players reported feeling that excessive criticism from coaches led to a decline in their confidence levels. In such circumstances, the cause may lie in coaches not always interacting effectively with former players or failing to employ appropriate communication methods. The pride displayed by coaches can instil pride in players, while the shame they project can evoke shame in players, further impacting athletes’ sporting performance [99]. Therefore, alongside fully recognising the injury characteristics of the sport and implementing sound training principles to gauge workload and enhance performance [100], coaches should also heighten their awareness and knowledge regarding athletes’ mental well-being. They must actively observe athletes’ psychological states during training and competition, as Forsberg et al. [101] indicate that performance in matches can influence multiple psychological states the following day.

## 6. Conclusions

This review synthesises evidence on common sports injuries in youth ice hockey players. Concussions and upper/lower limb injuries pose significant health threats. The aetiology is multifactorial, involving intrinsic (e.g., age, gender, injury history) and extrinsic factors (e.g., body checking rules, competition level, protective equipment).

Strong evidence supports modifying extrinsic risk factors as the most effective immediate prevention strategy. Implementing no-body-checking rules, enforcing fair play, and adopting zero-tolerance policies for dangerous play significantly reduce injury and concussion rates. Furthermore, properly fitted, sport-specific protective equipment—including full-face helmets and mouthguards—is crucial for mitigating injury severity.

Despite advances in understanding and prevention, numerous limitations persist. Future researchers should focus on developing age-, gender-, and developmental-specific injury assessment models and prevention strategies that fully account for individual differences such as sex, physiological maturity, skill level, and player position; establishing safety standards for youth-specific equipment; and enhancing injury prevention awareness and mental health among athletes and coaches. These measures are essential for safeguarding youth athletes’ health and promoting their sporting career well-being.

In summary, safeguarding the health of youth ice hockey players and fostering their long-term development necessitates the sustained implementation of evidence-based, multi-dimensional preventative measures. This demands collaborative efforts among researchers, sports governing bodies, coaches, clinicians, and parents to collectively cultivate safer sporting environments—maximising enjoyment while minimising risks—thereby ensuring the sustainable development of ice hockey.

## Figures and Tables

**Table 1:** Prevention Strategies for Common Sports Injuries in Teenage Ice Hockey Players.

Prevention Strategy	Concrete Measure	Core Findings/Analysis	Evidence Level (Based on Oxford CEBM Standards)
1. Fair Play and Rule Amendments	Establish a Fair Play Points (FPP) reward and penalty system; Combine FPP with the no-contact rule; Physical contact is strictly prohibited for players under the age of 12; All dangerous moves (such as back attacks) are strictly prohibited for all teenage players; Strict measures such as expulsion and suspension for dangerous behavior.	Can significantly reduce the risk of injury; Resulting in a significant reduction in rear-end collisions and frontal collisions; The incidence of concussions caused by direct lateral head impact was significantly reduced.	Level 2
2. Research and Development of Protective Equipment	Wear standard protective gear (helmet, shoulder pads, elbow pads, knee pads, etc.); Periodically check equipment integrity, paying attention to correct installation, shape and fit; New impact-resistant materials (such as D3O) can be used; Use integrated smart sensors to monitor acceleration, angular velocity, and other parameters in real time.	Scientific and reasonable equipment can effectively buffer the impact force, reduce the probability and severity of injury; Improper installation of equipment will weaken the protective function; The smart helmet accurately records head impact parameters, providing a basis for early detection of concussions.	Level 4
3. Specialised preparatory activities and neuromuscular training	Implement a neuromuscular training program based on the RAMP principle; Enhance muscle strength and flexibility in vulnerable areas (hip joint, adductor group); Strengthen core stability training; Join the Neck Control and Endurance Training module; Combine neuromuscular training with hockey-specific techniques.	It effectively reduces the risk of injury caused by neuromuscular control deficiency, optimizes biomechanical performance, reduces landing impact force, and improves sports performance; The solution has good applicability and acceptance.	Level 1
4. Education and management	Provide comprehensive safety and rules training for athletes and coaches; Focus on enhancing adolescents’ self-protection awareness, emphasizing the importance of wearing protective gear and thorough warm-up; The coach actively observes the athletes’ psychological state during training and competition.	While secondary concussion prevention education programs can improve cognitive understanding and safety awareness, the existing evidence base remains insufficient, and athletes struggle to translate knowledge into action; Excessive criticism from coaches can undermine players’ confidence; Performance in the match will affect a variety of psychological states the next day.	Level 4

## Data Availability

Not applicable.

## Abbreviations

TFCC	Triangular fibrocartilage complex
UCL	Ulnar collateral ligament
FFP	Full-face protection
PFP	Partial-face protection
NHL	National Hockey League
PIMS	Predetermined Penalty Minutes per Match
